# A case of neuroleptic malignant syndrome induced by olanzapine in postpartum period

**DOI:** 10.4103/0019-5545.37671

**Published:** 2007

**Authors:** Mehmet Üstündağ, Murat Orak, Cahfer Güloğlu, Mustafa Burak Sayhan, Mahmut Taş

**Affiliations:** Department of Emergency Medicine, Dicle University, Faculty of Medicine, Turkey

**Keywords:** Emergency department, neuroleptic malignant syndrome, olanzapine, postpartum period

## Abstract

Neuroleptic malignant syndrome (NMS) is a life-threatening medical complication that occurs as a result of dopaminergic receptor blockage in nigrostriatal pathways. This syndrome is mainly accepted to be an idiosyncratic reaction for antipsychotic medications. Incidence of NMS induced by olanzapine - an atypical antipsychotic - is extremely rare. However, there has been contradiction on postpartum period as a risk factor for NMS. This case is of interest due to the fact that it happens on postpartum period and is induced by olanzapine. We aimed in this study to evaluate the successfully cured case of neuroleptic malignant syndrome induced by olanzapine in postpartum period with the literature view.

## INTRODUCTION

Neuroleptic malignant syndrome (NMS) is a life-threatening condition that occurs as a result of dopaminergic receptor blockage in nigrostriatal pathways.[1] The main clinical symptoms of this syndrome are hyperthermia, muscle rigidity, autonomic instability and delirium.[2] Nonspecific laboratory findings are elevated CPK values, hyperkalemia and hypernatremia. Hypernatremia develops secondary to total body water loss. Death may occur due to cardiovascular collapse, renal or respiratory insufficiency and dysrythymia.[2] This syndrome is mainly accepted to be an idiosyncratic reaction for antipsychotic medications.[3] The atypical antipsychotics have different side effects than the conventional antipsychotic.

Although it has been frequently reported that this syndrome has been seen within the rates of 0.02-2.44% after classical antipsychotic medications,[4] the rates of NMS after atypical antipsychotic medications have not yet been described.[5] Olanzapine is a new generation thienobenzodiazepin antipsychotic having multiple receptor activity. It has effects on muscarinic, alpha-adrenergic, potent antiserotonergic (5HT2 and 5HT6) and antidopaminergic (D1-D5) receptors.[6] Because of this unique characteristic, the incidence of side effects of olanzapine, such as NMS and extrapyramidal symptoms, is extremely low.[7]

## CASE REPORT

A 20-year-old woman with schizophrenia diagnosis by a psychiatrist had been started a medication of oral olanzapine 10 mg/day. The patient had continuously used the drug for 16 months and has given up for 8 months because of pregnancy. During this period, the patient had not used any kind of medication and had not showed any psychotic symptoms. The patient had given birth to a healthy infant via normal vaginal way. However, 3 days after the parturition, the patient had started to nonsensical speaking and unconscious behavior, and psychiatrist had re-started oral olanzapine 20 mg/day. On the 10th day of the medication, the patient was admitted to our emergency clinics with the symptoms of stupor, high fever and muscle rigidity on arms, legs, jaw and feet.

No documentation of the patient was available in her history except for schizophrenia diagnosed 2 years before.

In physical examination, patient was lethargic, mean arterial blood pressure was 120/80 mmHg, pulsation was 160 beat/min, respiration rates were 20 breath/min and auxiliary temperature was 41°C. The pupils were isochoric and pupillary light reflexes were bilaterally normal. Tongue was dry and skin turgor tonus was decreased. Muscle rigidity could be seen in all extremities and the jaw. The patient had normal pulses in all the four extremities.

Auscultation of the lungs revealed the normal on both sides. Babinski reflex was also bilaterally negative. Glasgow coma scale of the patient was 11. The abdominal examination was unremarkable for acute findings or evidence of surgical scars.

Laboratory evaluation included an initial serum creatinine phosphokinase level of 5121.25 U/L. Leukocyte count was 5.700/uL with hemoglobin of 11 g/dL and a hematocrit of 38.7%. The patient's serum sodium level was 171.05 mmol/L, potassium 4.48 mmol/L, chloride 141.22 mmol/L, a blood urea nitrogen level 110 mg/dL and a creatinine of 2.18 mg/dL. The patient's serum lactic dehydrogenase level was 1409.39 U/L and sedimentation rate 19 mm/h. In arterial blood gas analyses, pCO_2_ was 27.2 mmHg, pO_2_ was 69.6 mmHg, sO_2_ 93.3% and pH was 7.477. No characteristics on ECG except for sinus tachycardia had been determined.

The results of analyses on cerebra-spinal fluid gathered by lumbar puncture were normal. The patient had neither meningitis nor encephalitis. CT of the brain was unremarkable. All blood cultures, urine cultures and cerebrospinal fluid cultures were negative for growth. According to the clinical and laboratory findings, the patient was diagnosed NMS and olanzapine treatment has been immediately cut-off.

After the diagnosis, the patient was taken to the intensive care unit for supportive care, a naso-gastric tube and urine catheter was replaced to the patient. Patient's respiration was sufficient so she did not intubated. A central venous catheter was also replaced to the subclavian vein. Central venous pressure was 0 cm/water. For the control of central venous pressure, during the first 24 h of admission, we have used 6000 mL of 5% dextrose. Total daily urine output was 4700 mL and after the fluid therapy, central venous pressure has been determined 4 cm/water, at the end of first day. Cold compresses were performed, methamizole sodium total 1 g TID was given intravenously and total 2.5 mg bromocriptin mesylate was given via naso-gastric tube. On the 48th hour of the treatment, serum sodium, urea and creatinine concentrations were 155 mmol/L, 85 mg/dL and 1.3 mg/dl, respectively. Fever had begun to decrease and Glasgow coma scale had risen to 15. Cold compresses and methamizole sodium treatment was stopped on the same treatment day. Parenteral fluid therapy has been continued, until central venous pressure has risen to 6-8 cm/water. Oral feeding has also been started, as the patient become conscious after the second day of the treatment. Serum CPK and LDH concentrations have begun to decrease after the third day. Major symptoms had been disappeared 5 days after the neuroleptic drug has been stopped. At the end of the treatment, physical examination and laboratory findings all revealed normal and the patient has been discharged from the hospital totally cured.

## DISCUSSION

Incidence of NMS side effect induced by olanzapine is extremely rare.[7] In literature research, totally 20 cases of NMS induced by olanzapine have been reported.[5] Considering these facts, our case of NMS occurred after olanzapine treatment is of interest. NMS is a serious side effect of antipsychotic medications.[8] It has usually been seen in young or middle-aged adults; however, it may occur with in 10 days after antipsychotic drug use and also at any phase of the treatment.[9] John *et al.*[3] have reported that in first 2 weeks, the occurrence rate is 80%. In this case, our patient was 20 years old and NMS has been seen on the 10th day of the olanzapine treatment.

Hyperthermia, muscle rigidity, autonomic instability, delirium and increased CPK values are the main symptoms of NMS.[2] Levenson[10] has reported that 52 of the 53 cases had hyperthermia. Hyperthermia (41°C) in our case last for 2 days and had not related with any other reasons. Furthermore, the patient had muscle rigidity on the arms, legs, jaw, hands and feet. She was lethargic during 48 h.

Laboratory findings in NMS are increase in creatinine concentrations, uremia, hypernatremia and hyperkalemia. Hypernatremia in NMS is secondary to the total body fluid loss.[2] In our case, increase in creatinine concentration, uremia and hypernatremia has been seen; however, hyperkalemia has not been determined.

Dehydration and nutrition disorders are the main risk factors to NMS.[11] Basic findings demonstrating dehydration, in this case increasing NMS risk factor, are those that the patient is in early postpartum period, has not been orally fed for 5 days, 0 cm/water of central venous pressure, 171.05 mmol/L of serum sodium concentration, dry appearance of the tongue and the decrease of skin turgor.

There has been contradiction on postpartum period as a risk factor for NMS. In literature, four cases of NMS occurred a few weeks after postpartum induced by neuroleptics has been documented.[12] This case is interesting with the facts that it happens on postpartum period and is induced by olanzapine.

Neuroleptic malignant syndrome is the deathly complication of neuroleptic medications.[2] Death usually occurs as a reason of cardiovascular collapse, renal or respiratory insufficiency and dysrhythmias.[2] In this case, with the early diagnosis and appropriate treatment, none of the deathly complications, such as cardiovascular collapse and renal or respiratory insufficiency, has been occurred. Major symptoms had been disappeared 15 days after the neuroleptic drug has been stopped and physical examination and laboratory findings all revealed normal. The patient has been completely cured and discharged from the hospital.

## CONCLUSION

The patients with NMS should admit to emergency services. In differential diagnosis of the patients referred to emergency services with the complaints of muscle rigidity, high fever, unconsciousness and antipsychotic drug use in history, NMS should also be considered. Although it is rare, practitioners need to be aware of that NMS may occur after olanzapine treatment. With early diagnosis and appropriate treatment, NMS that may cause death should be managed successfully.
